# The association between Monocyte‐to‐Lymphocyte ratio and postoperative delirium in ICU patients in cardiac surgery

**DOI:** 10.1002/jcla.24553

**Published:** 2022-06-16

**Authors:** Xunling Su, Jie Wang, Xing Lu

**Affiliations:** ^1^ Department of Anesthesiology zhejiang hospital Hangzhou China; ^2^ Department of Endocrinology Affiliated Hospital of Yanbian University Yanji China

**Keywords:** inflammatory markers, medical information Mart for intensive care‐III, monocyte‐to‐lymphocyte ratio, postoperative delirium

## Abstract

**Objective:**

To analyze the relationship between monocyte‐to‐lymphocyte ratio (MLR) and postoperative delirium (POD).

**Methods:**

This cohort study was conducted in the Medical Information Mart for Intensive Care‐III (MIMIC‐III) version 1.4 database. MLR was measured according to the complete blood count. ICD‐9 was used to measure postoperative delirium. Multivariable logistic regression was utilized to examine the relationship between MLR and POD.

**Results:**

Three thousand eight hundred sixty‐eight patients who had received cardiac surgery were retrospectively enrolled, including 2171 males and 1697 females, with a mean age of 63.9 ± 16.2 years. The univariate analysis suggested that high MLR (as a continuous variable) as associated with a 21% higher risk of POD (O R: 1.12, 95% CI, 1.02, 1.43, *p* = 0.0259), After adjustments for other confounding factors, gender, age, race, temperature, SBP, DBP, MAP, respiratory rate, SOFA, peripheral vascular disease, AG, psychoses, drug, and alcohol addiction, the results showed that high MLR (as a continuous variable) independently served as a risk factor for POD (OR: 1.21; 95% CI: 1.01–1.44; *p* = 0.0378). MLR was assessed as quintile and tertiles, high MLR was an independent risk factor for POD. In the subgroup analysis, there were no differences in MLR for patients with POD in pre‐specified subgroups.

**Conclusions:**

Monocyte‐to‐lymphocyte ratio was a risk factor for POD. More research is necessary to thoroughly examine the function of MLR in POD.

## Graphical abstract

Delirium, which is defined as an acute confessional condition whose characteristics include impaired mental state, is correlated with significant economic costs, high incidence of falls and fall‐related accidents, self‐removal of medical equipment, long‐term hospitalizations, and even increased fatality rates. Our study observed that patients suffering from POD showed significantly higher MLR patients without POD. Another important finding is that elevated MLR level independently served as a risk factor to predict POD.

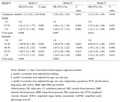

## 1 INTRODUCTION

Delirium, which is defined as an acute confessional condition whose characteristics include impaired mental state, is correlated with significant economic costs, high incidence of falls and fall‐related accidents, self‐removal of medical equipment, long‐term hospitalizations, and even increased fatality rates.^1^, ^2^ The incidence of postoperative delirium (POD) in heart surgery patients ranges from 50 to 70 percent. According to many prospective studies^3^, ^4^, ^5^, POD is correlated with significant short‐term repercussions, such as longer stays in the intensive care unit (ICU) and the hospital, as well as higher post‐surgical morbidity and death.^6^ Furthermore, POD has an influence on long‐term cognitive and functional deterioration, resulting in an increased demand for health care resources and expenses.^7^

Even though the exact mechanism behind the development of POD is not known at this time, neuroinflammation generated by the surgery‐induced systemic inflammatory process has been suggested to participate in this condition.^8^, ^9^, ^10^ While an elevated level of c‐reactive protein and interleukin‐6 (IL‐6) has also been shown to be correlated with POD,^11^ whereas one cohort research found no correlation between the level of plasma IL‐6 and delirium in elderly hospitalized patients. Consequently, the relationship between plasma markers of inflammation and POD is still unclear.^12^ POD might possibly be predicted using biomarkers associated with inflammation.^13^ It has been shown that the aberrant elevation in inflammation blood cell variables, including the neutrophil count, neutrophil‐to‐lymphocyte ratio, and platelet‐to‐lymphocyte ratio, functions as a basic indicator of inflammatory response; all of these parameters have been evaluated for their potential in POD.^14^, ^15^, ^16^ However, the predictive value of inflammatory biomarkers in POD has not been investigated independently and remains unknown.

Hemogram derived inflammatory markers have been reported to be associated with outcome in ICU population. These include platelet‐to‐lymphocyte count ratio ^17^, neutrophil‐to‐lymphocyte count ratio,^18^ and mean platelet volume.^19^ Therefore, another hemogram derived marker, monocyte‐to‐lymphocyte ratio (MLR), could be associated with worse outcome in ICU patients after cardiac surgery. The MLR is a newly developed and integrative inflammatory biomarker that is based on monocyte and lymphocyte counts^20^, ^21^. The increased MLR was initially used to assess the diabetic kidney injury^22^,liver steatosis,^23^ irritable bowel syndrome,^24^ cancer,^25^ and Covid‐19 infection ^26^; MLR is currently believed to better represent the inflammatory state of patients. The function of MLR and POD, on the other hand, remains controversial. We hypothesized that individuals with POD who had greater levels of inflammation, as evaluated by MLR, are at an elevated risk of developing POD. To investigate if MLR levels have a role in predicting POD, the present research examined the relationship between POD and MLR levels while controlling for a broad variety of possible confounders.

## 2 METHODS

### 2.1 Source of data and sample

We conducted a single‐center retrospective cohort study; we collected all relevant data from Medical Information Mart for Intensive Care‐III (MIMIC‐III Version 1.4)^27^, ^28^, ^29^ database. MIMIC‐III database is an open and freely available database developed by the Maslach‐setts Institute of Technology (MIT) computational Physiology Laboratory. The database records clinical medical information on patients admitted to the ICU at Beth Israel Deacon Medical Center from 2001 to 2012. The recorded information in the database included basic information, vital signs, supplementary tests, medication status, and diagnosis. The institutional Review Board of MIT and Beth Israel Deacon Medical Center granted approval to this database. The database is available to researchers who obtain a certificate after completing an online course on protecting Human Study Subjects, organized by the National Institutes of Health. Informed consent of the patients was exempted because the current study was a clinical database‐related study.

### 2.2 Selection criteria

Among the more than 50,000 different patients in the database, the subjects included in this study had to meet the following criteria: (1) The current procedural terminology (CPT) was utilized to identify patients undergoing cardiac surgery: Ideally, the CPT number ought to fall between 33,010 and 37,799, and (2) age ≥16 years. Exclusion criteria of this study were as follows: (1) younger than 16 years of age; (2) monocytes and lymphocyte count data were lost on the first day of admission to ICU; (3) data were missing by more than 20%; (4) intubated patients; and (5) patients had a hematologic neoplasm diagnosis.

### 2.3 Evaluation of MLR

The blood count was recorded, which included the absolute numbers of lymphocytes and monocytes. In addition, MLR was defined as MLR = M/L.

### 2.4 Assessment of outcome

Delirium within a hospitalization was defined by International Classification of Diseases‐ninth (ICD‐9) code. ICD‐9‐CM diagnostic code “2930,” “2931,” “29281,” “29011,” “2903,” “29041,” “2910,” “2939,” “78009,” “29381,” “29382,” “29383,” “29384,” “29389,” “29012,” “29013,” “29043,” “29211,” “29212,” “2922,” “78002,” “2902,” “29042,” “2908,” “2909,” “2920,” “29282,” “3483,” “34831,” “34839,” “34982,” and “78097.”

### 2.5 Baseline variables

Data were extracted by structured query language PostgreSQL 9.6. Demographic variables such as age, gender, race, and complications included hypertension, arrhythmia, heart valve disease, congestive heart failure, pulmonary circulation disorders, peripheral vascular disease, diabetes were acquired. All vital signs, results of blood gas (anions gap, lactate, and bicarbonate concentrations), lab findings, mechanical breathing time, and perioperative transfusion data were obtained. The sequential organ failure assessment (SOFA) score and s simplified acute physiology score II (SAPS II) were all computed while being admitted to the ICU.

### 2.6 Statistical Analysis

To verify normality, the Kolmogorov–Smirnov test and the Shapiro–Wilk test were performed. For normally distributed data, continuous variables are expressed as means ± standard deviations, whereas non‐normal distributed data are expressed as median ± interquartile range. Numeric values (%) are used to express categorical variables. Statistical comparisons were carried out utilizing the Mann–Whitney U‐test in the case of continuous data whereas the two‐tailed t‐test or Fisher exact test were utilized in the case of categorical data. To evaluate the relationship between MLR and all outcomes, a univariate logistic regression analysis was performed. Subsequently, a multivariate logistic regression analysis was carried out for the purpose of calibrating the subsequent important covariates. The odds ratio (OR) was calculated with a 95 percent confidence interval (CI) to represent the effect. We also made adjustments for the factors that were correlated with the dominant and secondary outcomes. In addition, multivariate analysis was used to control for the corresponding confounding factors; in model I, the confounding factors, including age, gender, and race were adjusted, while in model II, confounding factors, including age, sex, race, diastolic blood pressure, heart rate, respiratory rate, temperature, SpO2, heart failure anion gap, platelet, serum chloride. Were adjusted. Furthermore, subgroup analyses were conducted in order to corroborate the validity of our results.

STATA (version: 15.0) (STATA Corp LLC) was employed to conduct the statistical analyses. Statistical significance was considered to have been attained when *p* < 0.05.

## 3 RESULTS

### 3.1 Patient characteristics

A total of 3868 patients with cardiac surgery were retrospectively enrolled, including 2171 males and 1697 females, with a mean age of 63.9 ± 16.2 years. According to the absence or presence of POD, the patients were classified into POD and non‐POD groups; data for a sum of 562 patients in the POD cohort and 3306 in the non‐POD cohort were analyzed. The patient baseline information is shown in Table 1. The patients in the POD group had significantly higher SOFA scores, SAPS II scores, Elixhauser comorbidity index scores, and MLR as compared to those in the POD group. Vital signs, including mean heart rate, respiration rate, temperature, SBP, DBP, and MAP were higher in the POD group, Laboratory indicators were used for the assessment of organ functions for both groups of patients. The results demonstrated that there was no significant difference between the two groups. Patients in the Pod group had higher rates of drug and alcohol addiction. We further compared three groups based on MLR, and the patient baseline information shown in Table 2. As opposed to the patients in the low MLR group, those in the high MLR group are dominantly female, the white race, elevated CHF rate, arrhythmia, delirium, hypertension, kidney failure disease, greater platelet, and WBC counts, elevated heart rate, PT, APTT, anion gap, BUN, creatinine, lactate, and respiratory rate, reduced levels of SPO2, chloride, bicarbonate, MAP, hemoglobin, and elevated SOFA scores, SAPS II scores, Elixhauser comorbidity index scores.

**TABLE 1:** Baseline characteristics of the study population

Characteristics	Non‐POD	POD	*p* value
*N*	3306	562	
Age, years	64.0 ± 16.1	64.1 ± 17.0	0.833
Gender, *n* (%)			
Female	1456 (44.0)	241 (42.9)	0.609
Male	1850 (56.0)	321 (57.1)	
Ethnicity, *n* (%)			
White	2361 (71.4)	411 (73.1)	0.695
Black	345 (10.4)	54 (9.6)	
Other	600 (18.1)	97 (17.3)	
Vital signs			
Heart rate, beats/min	89.2 ± 16.7	91.3 ± 17.5	0.004
SBP, mmHg	114.8 ± 16.1	118.1 ± 17.8	**<0.001**
DBP, mmHg	59.4 ± 10.5	61.9 ± 11.3	**<0.001**
MAP, mmHg	76.2 ± 10.8	78.2 ± 12.0	**<0.001**
RR, times/min	19.9 ± 4.4	20.6 ± 4.8	**0.003**
Temperature, °C	36.8 ± 0.7	36.9 ± 0.8	**0.010**
SpO2, %	97.2 ± 2.4	97.1 ± 2.3	0.636
Comorbidities, *n* (%)			
CS	174 (5.3)	21 (3.7)	0.126
CHF	1089 (32.9)	199 (35.4)	0.251
Cardiac arrhythmias	1105 (33.4)	201 (35.8)	0.278
PCD	309 (9.3)	56 (10.0)	0.643
Valvular disease	466 (14.1)	77 (13.7)	0.803
PVD	375 (11.3)	86 (15.3)	**0.007**
Hypertension	1872 (56.6)	335 (59.6)	0.186
Diabetes	740 (22.4)	112 (19.9)	0.194
Renal failure	680 (20.6)	133 (23.7)	0.096
Drug abuse	113 (3.4)	51 (9.1)	**<0.001**
Alcohol abuse	256 (7.7)	87 (15.5)	**<0.001**
Laboratory parameters			
MLR (%)	60 ± 100	60 ± 90	**<0.001**
Anion gap, mmol/L	16.9 ± 5.5	17.4 ± 5.5	**0.028**
Albumin, mg/dl	3.1 ± 0.7	3.1 ± 0.7	0.636
Bilirubin, mg/dl	2.5 ± 5.4	2.0 ± 4.3	0.115
Creatinine, mg/dl	2.0 ± 2.0	2.1 ± 2.6	0.098
Chloride, mmol/L	108.1 ± 7.1	108.1 ± 7.4	0.981
Maximum glucose, mg/dl	192.2 ± 103.8	190.9 ± 102.1	0.793
Mean glucose, mg/dl	143.3 ± 44.7	144.4 ± 47.7	0.577
WBC, 10^9^/L	13.6 ± 11.1	13.1 ± 7.7	0.285
Monocyte, %	4.3 ± 3.5	4.5 ± 3.5	0.020
Hematocrit, %	35.5 ± 6.1	35.5 ± 6.4	0.958
Hemoglobin, g/dl	11.8 ± 2.1	11.8 ± 2.2	0.825
Band neutrophils, %	11.0 ± 11.2	11.0 ± 11.9	0.969
Lactate, mmol/L	3.4 ± 2.8	3.1 ± 2.6	**0.043**
Bicarbonate, mmol/L	24.6 ± 5.0	24.8 ± 5.0	0.234
Sodium, mmol/L	140.5 ± 5.2	140.8 ± 5.4	**0.045**
Potassium, mmol/L	4.9 ± 1.0	4.8 ± 1.0	0.254
APTT, second	49.0 ± 33.3	45.3 ± 29.8	**0.017**
PT, second	18.6 ± 11.4	19.0 ± 14.5	0.487
INR	1.8 ± 1.7	1.8 ± 1.7	**0.027**
BUN, mg/dl	35.1 ± 27.5	37.3 ± 29.5	0.085
Scoring system			
ECI	18.6 ± 14.0	21.7 ± 15.6	**<0.001**
SOFA	6.2 ± 3.8	6.5 ± 3.6	**0.024**
SAPSII	42.4 ± 14.8	43.9 ± 14.6	**0.027**
Hospital Los, days	15.2 ± 13.8	22.0 ± 17.9	**<0.001**

Abbreviations: APTT, activated partial thromboplastin time; BUN, blood urea nitrogen; CHF, congestive heart failure; CS, cardiac shock; DBP, diastolic blood pressure; ECI, Elixhauser comorbidity index; INR, international normalized ratio; Los, length of stay; MBP, mean blood pressure; PCD, pulmonary circulation disease; PT, prothrombin time; PVD, peripheral vascular disease; RR, respiration rate; SAPSII, simplified acute physiology score II; SBP, systolic blood pressure; SOFA, sequential organ failure assessment; SpO2, pulse oximetry‐derived oxygen saturation; WBC, white blood cell.

Bold values represent *p* 〈 0.05

**TABLE 2:** Baseline characteristics of the study population

Characteristics	Monocyte‐to‐lymphocyte ratio(%)			*p* value
	<27	27–53	53	
*N*	1288	1288	1292	
Admission age, years	63.3 ± 16.2	64.1 ± 16.2	64.5 ± 16.3	0.159
Gender, *n* (%)				
0	589 (45.7)	583 (45.3)	525 (40.6)	**0.016**
1	699 (54.3)	705 (54.7)	767 (59.4)	
Ethnicity, *n* (%)				
0	866 (67.2)	937 (72.7)	969 (75.0)	**<0.001**
1	166 (12.9)	144 (11.2)	89 (6.9)	
2	256 (19.9)	207 (16.1)	234 (18.1)	
Vital signs				
Heartrate, beats/min	87.9 ± 16.2	88.3 ± 16.3	92.2 ± 17.6	**<0.001**
SBP, mmHg	115.8 ± 15.8	116.7 ± 16.9	113.4 ± 16.4	**<0.001**
DBP, mmHg	59.9 ± 10.2	60.5 ± 11.1	58.9 ± 10.6	**<0.001**
MBP, mmHg	77.0 ± 10.7	77.2 ± 11.4	75.3 ± 10.8	**<0.001**
RR, times/min	19.4 ± 4.4	20.1 ± 4.3	20.6 ± 4.6	**<0.001**
Temperature, °C	36.8 ± 0.7	36.9 ± 0.7	36.9 ± 0.7	0.252
SpO2, %	97.4 ± 2.5	97.1 ± 2.4	97.0 ± 2.3	**<0.001**
Comorbidities, *n* (%)				
Delirium	150 (11.6)	206 (16.0)	206 (15.9)	**0.002**
CS	50 (3.9)	65 (5.0)	80 (6.2)	**0.027**
CHF	375 (29.1)	466 (36.2)	447 (34.6)	**<0.001**
Cardiac arrhythmias	399 (31.0)	438 (34.0)	469 (36.3)	**0.016**
PCD	116 (9.0)	133 (10.3)	116 (9.0)	0.409
Valvular disease	183 (14.2)	186 (14.4)	174 (13.5)	0.759
PVD	166 (12.9)	161 (12.5)	134 (10.4)	0.105
Hypertension	775 (60.2)	733 (56.9)	699 (54.1)	**0.008**
Diabetes	295 (22.9)	272 (21.1)	285 (22.1)	0.550
Renal failure	231 (17.9)	286 (22.2)	296 (22.9)	**0.004**
Drug abuse	53 (4.1)	62 (4.8)	49 (3.8)	0.421
Alcohol abuse	85 (6.6)	132 (10.2)	126 (9.8)	**0.002**
Laboratory parameters				
MLR	0.2 ± 0.1	0.4 ± 0.1	1.3 ± 1.4	**<0.001**
Anion gap, mmol/L	15.8 ± 5.4	17.1 ± 5.5	17.9 ± 5.6	**<0.001**
Albumin, mg/dl	3.2 ± 0.7	3.1 ± 0.7	3.0 ± 0.7	**<0.001**
Bilirubin, mg/dl	1.6 ± 3.5	2.3 ± 5.4	3.0 ± 6.1	**<0.001**
Creatinine, mg/dl	1.7 ± 2.1	2.1 ± 2.2	2.2 ± 2.1	**<0.001**
Chloride, mmol/L	109.6 ± 6.5	107.6 ± 6.9	107.2 ± 7.6	**<0.001**
Maximum glucose, mg/dl	196.9 ± 106.6	187.3 ± 102.7	191.9 ± 101.1	0.063
Mean glucose, mg/dl	141.1 ± 44.6	143.6 ± 44.7	145.7 ± 46.2	0.035
WBC, 10^9^/L	11.4 ± 7.4	12.6 ± 6.7	16.4 ± 15.1	**<0.001**
Monocyte, %	2.9 ± 1.7	4.1 ± 2.1	5.9 ± 5.0	**<0.001**
Hematocrit, %	36.2 ± 5.9	35.2 ± 6.1	35.2 ± 6.4	**<0.001**
Hemoglobin, g/dl	12.0 ± 2.1	11.7 ± 2.1	11.6 ± 2.2	**<0.001**
Band neutrophils, %	12.3 ± 13.5	10.4 ± 10.2	10.8 ± 10.8	0.091
Bicarbonate, mmol/L	24.8 ± 4.2	24.9 ± 5.1	24.2 ± 5.5	**0.002**
Lactate, mmol/L	3.4 ± 2.7	3.2 ± 2.7	3.5 ± 2.9	**0.038**
Sodium, mmol/L	141.0 ± 4.8	140.7 ± 5.1	139.9 ± 5.8	**<0.001**
Potassium, mmol/L	5.0 ± 1.1	4.8 ± 1.0	4.8 ± 1.0	**<0.001**
APTT, second	48.2 ± 32.4	47.8 ± 32.8	49.4 ± 33.4	**0.015**
INR	1.6 ± 0.9	1.9 ± 1.8	2.0 ± 2.1	**<0.001**
PT, second	17.3 ± 9.1	18.6 ± 12.7	20.1 ± 13.3	**<0.001**
BUN, mg/dl	29.8 ± 23.1	35.7 ± 29.1	40.9 ± 29.8	**<0.001**
Scoring system				
ECI	15.6 ± 14.2	19.2 ± 14.0	22.3 ± 13.8	**<0.001**
SOFA	5.8 ± 3.6	6.0 ± 3.6	6.9 ± 4.0	**<0.001**
SAPSII	40.3 ± 14.6	42.0 ± 14.7	45.4 ± 14.6	**<0.001**
Hospital Los, days	13.9 ± 13.3	16.3 ± 13.9	18.2 ± 16.4	**<0.001**

Abbreviations: APTT, activated partial thromboplastin time; BUN, blood urea nitrogen; CHF, congestive heart failure; CS, cardiac shock; DBP, diastolic blood pressure; ECI, Elixhauser comorbidity index; INR, international normalized ratio; Los, length of stay; MBP, mean blood pressure; PCD, pulmonary circulation disease; PT, prothrombin time; PVD, peripheral vascular disease; RR, respiration rate; SAPSII, simplified acute physiology score II; SBP, systolic blood pressure; SOFA, sequential organ failure assessment; SpO2, pulse oximetry‐derived oxygen saturation; WBC, white blood cell.

Bold values represent *p* 〈 0.05

### 3.2 Relationship between MLR and POD

Following adjustments for the potential confounding variables, we developed distinct models for the purpose of evaluating the independent impacts of MLR on the POD. As shown in Table 3, the OR and 95% CI values and the univariate analysis suggested that high MLR(As a continuous variable) is associated with a 21% higher risk of POD (O R: 1.12, 95% CI, 1.02, 1.43, *p* = 0.0259), After adjustments for other confounding factors, gender, age, race, temperature, SBP, DBP, MAP, respiratory rate, SOFA, peripheral vascular disease, AG, psychoses, drug, and alcohol addiction, the results showed that high MLR (As a continuous variable) independently served as a risk factor to predict POD (OR: 1.21; 95% CI: 1.01–1.44; *p* = 0.0378).

**TABLE 3:** ORs (95% CIs) for POD across groups of MLR

MLR (%)	Model 1^a^		Model 2^b^		Model 3^c^	
	OR (95% CIs)	*p* value	OR (95% CIs)	*p* value	OR (95% CIs)	*p* value
Continuous variable 1.21 (1.02, 1.43) 0.026			1.20 (1.02, 1.42)	0.031	1.21 (1.01, 1.44)	0.038
Tertile						
<27	1.0		1.0		1.0	
27–53	1.41 (1.12, 1.77)	0.003	1.40 (1.12, 1.76)	0.004	1.33 (1.04, 1.69)	0.022
>53	1.45 (1.15, 1.82)	0.001	1.44 (1.15, 1.81)	0.002	1.41 (1.11, 1.80)	0.005
*p* for trend	0.006		0.007		0.014	
Quintiles						
<19	1.0		1.0		1.0	
19–30	1.09 (0.79, 1.49)	0.601	1.09 (0.79, 1.49)	0.603	0.99 (0.71, 1.40)	0.976
30–50	1.66 (1.23, 2.22)	<0.001	1.65 (1.23, 2.22)	<0.001	1.59 (1.16, 2.18)	0.004
45–74	1.48 (1.10, 1.99)	0.010	1.47 (1.09, 1.98)	0.012	1.42 (1.03, 1.96)	0.030
≥74	1.57 (1.17, 2.11)	0.003	1.56 (1.16, 2.10)	0.003	1.51 (1.10, 2.07)	0.012
*p* for trend	0.004		0.004		0.009	

*Note*: Models 1, 2 and 3 were derived from logistic regression models.

Abbreviations: OR, odds ratio; CI, confidence interval; SBP, systolic blood pressure; DBP, diastolic blood pressure; MBP, mean blood pressure; RR, respiration rate; PVD, peripheral vascular disease; SOFA, sequential organ failure assessment; SAPSII, simplified acute physiology score II.

^a^ Model 1 covariates were adjusted for nothing.

^b^ Model 2 covariates were adjusted for age, sex and race.

^c^ Model 3 covariates were adjusted for age, sex, race, temperature, psychoses, PVD, alcohol abuse, drug abuse, RR, SOFA, MBP, SBP, DBP, and SAPSII.

When MLR was assessed as tertiles, we found that patients in high MLR (MLR ≥51) also had significantly higher risks of POD (OR 1.45, 95% CI 1.15–1.82, *p* = 0.0014) as opposed to patients in the low group (MLR <26) in the univariate model, After adjustments for other confounding factors, gender, age, race, temperature, SBP, DBP, MAP, respiratory rate, SOFA, peripheral vascular disease, AG, psychoses, drug, and alcohol addiction, the results showed that high MLR (MLR ≥51) independently served as a risk factor to predict POD (OR: 1.55; 95% CI: 1.09–2.19; *p* = 0.0138).

When MLR was assessed as quintile, we found that patients in high MLR (MLR ≥73) also had significantly higher risks of POD (OR 1.57, 95% CI 1.17–2.11, *p* = 0.0027) than patients in the low group (MLR <0.18) in the univariate model, After adjustments for other confounding factors, gender, age, race, temperature, SBP, DBP, MAP, respiratory rate, SOFA, peripheral vascular disease, AG, psychoses, drug and alcohol addiction, the results showed that high MLR (MLR ≥73) independently served as a risk factor to predict POD (OR: 1.51; 95% CI: 1.10–2.07; *p* = 0.0115).

### 3.3 subgroup analysis

The results of subgroup analysis are shown in Table 4. There were no differences in MLR for patients with POD in pre‐specified subgroups.

**TABLE 4:** Subgroup analysis of MLR and POD

Characteristic	*N*	Monocyte‐to‐lymphocyte ratio (%)			*p* for interaction
		27	27–53	53	
Age, years					
<65.4	1900	1.0	1.64 (1.18, 2.29)	1.69 (1.21, 2.36)	0.833
≥65.4	1910	1.0	1.21 (0.88, 1.66)	1.25 (0.91, 1.70)	
Gender, *n* (%)					
Female	1676	1.0	1.45 (1.04, 2.02)	1.22 (0.86, 1.74)	0.499
Male	2134	1.0	1.37 (1.00, 1.87)	1.62 (1.20, 2.18)	
Ethnicity, *n* (%)					
White	2726	1.0	1.48 (1.13, 1.95)	1.42 (1.08, 1.86)	0.493
Black	396	1.0	1.31 (0.68, 2.51)	1.12 (0.52, 2.42)	
Other	688	1.0	1.13 (0.64, 1.99)	1.73 (1.04, 2.89)	
Neutrophils, %					
<82.5	1910	1.0	1.69 (1.25, 2.28)	1.52 (1.09,2.11)	0.104
≥82.5	1900	1.0	1.11 (0.77, 1.59)	1.28 (0.90,1.81)	
Lymphocytes, %					
<9.6	1872	1.0	1.01 (0.66, 1.54)	0.96 (0.64, 1.43)	0.087
≥9.6	1938	1.0	1.52 (1.14, 2.01)	2.14 (1.45, 3.16)	
WBC, 10^9^/L					
<11.7	1909	1.0	1.48 (1.10, 2.00)	1.81 (1.32, 2.49)	0.876
≥11.7	1901	1.0	1.31 (0.92, 1.87)	1.24 (0.88, 1.74)	
SOFA					
<6	1869	1.0	1.70 (1.22, 2.38)	1.68 (1.19, 2.38)	0.124
≥6	1941	1.0	1.17 (0.85, 1.60)	1.23 (0.91, 1.66)	
SAPSII					
<41	1885	1.0	1.86 (1.35, 2.58)	1.53 (1.08, 2.18)	0.051
≥41	1925	1.0	1.03 (0.75, 1.42)	1.24 (0.91, 1.67)	
Heart rate, beats/min					
<88.3	1902	1.0	1.19 (0.86, 1.65)	1.44 (1.03, 2.00)	0.900
≥88.3	1898	1.0	1.61 (1.16, 2.22)	1.43 (1.04, 1.96)	
SBP, mmHg					
<112.1	1892	1.0	1.56 (1.09, 2.23)	1.66 (1.18, 2.34)	0.375
≥112.1	1903	1.0	1.27 (0.95, 1.72)	1.34 (0.99, 1.82)	
DBP, mmHg					
<58.8	1887	1.0	1.40 (1.00, 1.97)	1.24 (0.89, 1.74)	0.749
≥58.8	1907	1.0	1.36 (1.00, 1.86)	1.67 (1.23, 2.28)	
MBP, mmHg					
<74.95	1890	1.0	1.59 (1.13, 2.25)	1.41 (1.00, 1.98)	0.129
≥74.95	1910	1.0	1.25 (0.92, 1.70)	1.56 (1.15, 2.11)	
Temperature, °C					
<36.8	1825	1.0	1.54 (1.08, 2.19)	1.96 (1.39, 2.75)	0.030
≥36.8	1825	1.0	1.34 (0.98, 1.85)	1.14 (0.83, 1.59) 0	
RR, times/min					
<19.2	1910	1.0	1.50 (1.09, 2.07)	1.52 (1.09, 2.11)	0.504
≥19.2	1890	1.0	1.25 (0.90, 1.73)	1.32 (0.96, 1.81)	
SpO2, %					
<97.5	1889	1.0	1.40 (1.01, 1.94)	1.32 (0.95, 1.83)	0.148
≥97.5	1909	1.0	1.35 (0.97, 1.86)	1.58 (1.15, 2.17)	
Mean glucose, mg/dl					
<133.7	1910	1.0	1.64 (1.20, 2.25)	1.53 (1.10, 2.12)	0.668
≥133.7	1890	1.0	1.19 (0.85, 1.66)	1.35 (0.98, 1.85)	
Monocyte, %					
<3.6	1884	1.0	1.27 (0.93, 1.72)	1.30 (0.92, 1.82)	0.692
≥3.6	1926	1.0	1.57 (1.07, 2.30)	1.58 (1.09, 2.28)	
Anion gap, mmol/L					
<16	1739	1.0	1.71 (1.22, 2.40)	1.95 (1.37, 2.76)	0.051
≥16	2052	1.0	1.12 (0.82, 1.53)	1.09 (0.80, 1.47)	
Albumin, mg/dl					
<3.1	1068	1.0	1.47 (0.92, 2.34)	1.50 (0.96, 2.34)	0.489
≥3.1	1085	1.0	1.24 (0.82, 1.87)	1.20 (0.80, 1.81)	
Bilirubin, mg/dl					
<0.8	1280	1.0	1.57 (1.10, 2.24)	1.22 (0.83, 1.79)	0.701
≥0.8	1329	1.0	0.87 (0.57, 1.33)	1.22 (0.84, 1.78)	
Creatinine, mg/dl					
<1.2	1711	1.0	1.80 (1.27, 2.55)	1.96 (1.37, 2.80)	0.036
≥1.2	2096	1.0	1.12 (0.83, 1.52)	1.11 (0.83, 1.50)	
Chloride, mmol/L					
<108	1748	1.0	1.47 (1.03, 2.09)	1.25 (0.88, 1.79)	0.015
≥108	2060	1.0	1.32 (0.97, 1.79)	1.66 (1.23, 2.24)	
Maximum glucose, mg/dl					
<166	1899	1.0	1.48 (1.07, 2.05)	1.32 (0.94, 1.85)	0.221
≥166	1909	1.0	1.31 (0.95, 1.82)	1.58 (1.16, 2.16)	
Hematocrit, %					
<35	1886	1.0	1.42 (1.03, 1.96)	1.26 (0.91, 1.74)	0.132
≥35	1922	1.0	1.34 (0.96, 1.85)	1.65 (1.20, 2.26)	
Hemoglobin, g/dl					
<11.6	1863	1.0	1.42 (1.02, 1.96)	1.28 (0.92, 1.77)	0.423
≥11.6	1943	1.0	1.35 (0.98, 1.87)	1.62 (1.18, 2.22)	
Lactate, mmol/L					
<2.5	1605	1.0	1.38 (0.98, 1.94)	1.56 (1.12, 2.19)	0.977
≥2.5	1601	1.0	1.45 (1.01, 2.07)	1.34 (0.94, 1.90)	
Potassium, mmol/L					
<4.6	1720	1.0	1.34 (0.95, 1.87)	1.35 (0.96, 1.90)	0.572
≥4.6	2088	1.0	1.44 (1.05, 1.96)	1.52 (1.12, 2.06)	
APTT, second					
<35.7	1847	1.0	1.36 (1.00, 1.86)	1.40 (1.02, 1.93)	0.936
≥35.7	1857	1.0	1.39 (0.98, 1.97)	1.48 (1.06, 2.07)	
INR					
<1.4	1685	1.0	1.30 (0.94, 1.78)	1.29 (0.92, 1.80)	0.508
≥1.4	2021	1.0	1.51 (1.08, 2.12)	1.63 (1.18, 2.25)	
PT, second					
<15.4	1822	1.0	1.24 (0.91, 1.68)	1.16 (0.84, 1.61)	0.724
≥15.4	1884	1.0	1.62 (1.14, 2.31)	1.83 (1.31, 2.56)	
Sodium, mmol/L					
<140	1547	1.0	1.46 (0.99, 2.14)	1.37 (0.94, 1.99)	0.422
≥140	2261	1.0	1.38 (1.04, 1.83)	1.54 (1.16, 2.06)	
BUN, mg/dl					
<26	1855	1.0	1.87 (1.35, 2.58)	1.88 (1.34, 2.65)	0.153
≥26	1953	1.0	1.03 (0.74, 1.42)	1.09 (0.80, 1.49)	
Bicarbonate, mmol/L					
<25	1884	1.0	1.31 (0.93, 1.85)	1.46 (1.05, 2.02)	0.622
≥25	1921	1.0	1.49 (1.10, 2.02)	1.47 (1.07, 2.02)	
Band neutrophils, %					
<7	514	1.0	1.20 (0.61, 2.39)	1.41 (0.77, 2.57)	0.183
≥7	603	1.0	1.51 (0.80, 2.86)	0.97 (0.53, 1.77)	
CS					
No	3615	1.0	1.41 (1.12, 1.77)	1.46 (1.16, 1.85)	0.976
Yes	195	1.0	1.58 (0.45, 5.58)	1.41 (0.41, 4.84)	
CHF					
No	2545	1.0	1.45 (1.09, 1.91)	1.44 (1.09, 1.91)	0.890
Yes	1265	1.0	1.31 (0.88, 1.94)	1.43 (0.96, 2.11)	
Cardiac arrhythmias					
No	2527	1.0	1.40 (1.06, 1.85)	1.34 (1.01, 1.77)	0.419
Yes	1283	1.0	1.42 (0.95, 2.11)	1.65 (1.12, 2.43)	
PCD					
No	3448	1.0	1.38 (1.09, 1.76)	1.40 (1.10, 1.78)	0.711
Yes	362	1.0	1.65 (0.78, 3.51)	1.99 (0.93, 4.25)	
Valvular disease					
No	3273	1.0	1.37 (1.07, 1.75)	1.49 (1.16, 1.90)	0.450
Yes	537	1.0	1.66 (0.92, 3.00)	1.22 (0.65, 2.28)	
PVD					
No	3354	1.0	1.43 (1.11, 1.84)	1.52 (1.19, 1.94)	0.849
Yes	456	1.0	1.30 (0.74, 2.28)	1.18 (0.65, 2.14)	
Hypertension					
No	1638	1.0	1.30 (0.91, 1.86)	1.23 (0.86, 1.77)	0.604
Yes	2172	1.0	1.49 (1.11, 2.00)	1.64 (1.22, 2.20)	
Diabetes					
No	2969	1.0	1.38 (1.07, 1.77)	1.35 (1.04, 1.74)	0.528
Yes	841	1.0	1.52 (0.90, 2.58)	1.92 (1.16, 3.18)	
Renal failure					
No	3016	1.0	1.46 (1.12, 1.90)	1.60 (1.24, 2.07)	0.247
Yes	794	1.0	1.19 (0.74, 1.90)	0.99 (0.61, 1.59)	
Drug abuse					
No	3650	1.0	1.43 (1.12, 1.82)	1.51 (1.19, 1.91)	0.416
Yes	160	1.0	1.07 (0.48, 2.37)	0.98 (0.42, 2.33)	
Alcohol abuse					
No	3473	1.0	1.40 (1.10, 1.80)	1.46 (1.14, 1.87)	0.741
Yes	337	1.0	1.09 (0.58, 2.08)	1.07 (0.56, 2.04)	
ECI					
<18	1835	1.0	1.63 (1.17, 2.27)	1.45 (1.01, 2.07)	0.387
≥18	1973	1.0	1.16 (0.85, 1.60)	1.27 (0.94, 1.72)	

*Note*: ORs (95% CIs) were derived from logistic regression models. Covariates were adjusted as in model 1 (Table 3).

Abbreviations: APTT, activated partial thromboplastin time; BUN, blood urea nitrogen; CHF, congestive heart failure; CS, cardiac shock; DBP, diastolic blood pressure; ECI, Elixhauser comorbidity index; INR, international normalized ratio; MBP, mean blood pressure; PCD, pulmonary circulation disease; PT, prothrombin time; PVD, peripheral vascular disease; RR, respiration rate; SAPSII, simplified acute physiology score II; SBP, systolic blood pressure; SOFA, sequential organ failure assessment; SpO2, pulse oximetry‐derived oxygen saturation; WBC, white blood cell.

## 4 DISCUSSION

As far as we know, this study is the first to demonstrate the strong correlation between MLR and POD. We observed that patients suffering from POD showed significantly higher MLR patients without POD. Another important finding is that elevated MLR level independently served as a risk factor to predict POD.

Increasing research evidence suggests that neuroinflammation might perform a function in the progression of POD.^30^, ^31^ It has been shown that when the central nervous system's homeostasis is disrupted, a large number of inflammatory mediators produced by activated microglia cause neuroinflammation to occur.^32^, ^33^ Despite the fact that elevated levels of inflammatory cytokine have been correlated with the occurrence and progression of POD, measuring inflammatory markers is a costly procedure that cannot be performed in many institutions.^33^ However, since the inflammatory mediators used in the present research could be derived from the findings of a full blood count examination, they are simple to use and affordable to obtain. It has been shown that the MLR may function as a biomarker for both systematic inflammatory responses and neuroinflammation in individuals suffering from depression.^34^ It is common practice to utilize inflammatory markers found in regular blood testing to predict the prognosis of POD, and these indicators represent the extent of systemically low‐intensity inflammatory response. As a result, they are used to treat a broad range of clinical disorders in different settings.

Nevertheless, the link between MLR and POD remains unclear. Local inflammation in injured brain areas contributes to secondary brain injury by enhancing the disintegration of the blood–brain barrier, neural death, oxidative stress, cerebral edema, and microvascular failure that occurs due to the damage.^35^, ^36^ The inflammation could manifest itself throughout the brain, with long‐term consequences for the patient's cognitive functioning.^37^ The primary damage caused by POD delivers a chain of occurrences, such as the secretion of excitotoxic compounds, oxidative stress, and mitochondrial illnesses, all of which contribute to secondary brain injury, manifesting as compaction of brain tissue, impaired blood clotting, an intracellular biochemistry sequence reaction, inflammation, and other symptoms. POD induces a number of inflammatory responses to occur, as well as the activation of the immunological responses. In the CNS, the microglia are among the early cell types to react when these potentially lethal signals are received. Following injury, microglia become stimulated, experience structural changes, and release cytokines in just a few minutes. Various investigations have demonstrated that the mobilization and stimulation of monocytes may enhance the inflammatory response and exert a function comparable to that of microglia, elevating the expression of pro‐inflammatory factors as well as chemokines. We believe that our results will pave the way for a new line of study, including the examination of blood cell counts and inflammation metrics in POD patients in the future. Obviously, inflammatory ratios are low‐cost and readily available measures of inflammation, and that they may be obtained by a standard blood test. Furthermore, research has revealed that inflammatory ratios are significantly correlated with other recognized inflammatory indicators, including oxidative stress, and several pro‐inflammatory cytokines. Notably, these measures appear to be less impacted by exercise, catecholamine secretion, and other confounding variables compared to single leukocyte measurements or other regularly used indicators of inflammation.

There were some positive aspects to our research. Above all, the continual sampling of patients eliminates the possibility of selection bias. Secondly, the target‐independent factors are divided into three or five groups, which minimized the likelihood of error in data analysis and increases the robustness of the conclusions in the present research. Thirdly, repeated imputations were performed to analyze the incomplete data and ensure that the findings obtained from the whole data set and the numerous imputed datasets were congruent with one another.

Our research, on the other hand, has several drawbacks: (1) Causality cannot be established because of the retrospective observational study design. Prospective studies are needed to solve this. (2) The information was obtained from a single blood test. Serial testing might be more beneficial than a single test performed upon admission. (3) With respect to clinical practice, obtaining MLR is very simple; nonetheless, the absence of M and L in the database remains prevalent, resulting in selection bias.

## 5 CONCLUSION

In conclusion, we offered the first proof that MLR is correlated with an elevated chance of developing POD. MLR might be an accessible and reliable marker that can be used to predict POD in ICU patients in cardiac surgery. This finding should be confirmed in prospective studies.

## CONFLICT OF INTEREST

None.

## Supporting information


Table S1

## Data Availability

All the data used to support this study are available from the corresponding author (E‐mail: kapalu1979@sina.com) upon request.
