# Supplementary material for: The association between Monocyte‐to‐Lymphocyte ratio and postoperative delirium in ICU patients in cardiac surgery
Source: J Clin Lab Anal. 2022 Jun 16;36(7):e24553. doi: 10.1002/jcla.24553 (PMC9280003; doi:10.1002/jcla.24553)
Supplement: Supplementary file 1 — Table S1 [file JCLA-36-e24553-s001.docx]

| Characteristic |  | OR (95% CIs) *P* value |
| --- | --- | --- |
| Admission age, years | 64.00 ± 16.22 | 1.00 (1.00, 1.01) 0.833 |
| Gender, n (%) |  |  |
| 0 | 1697 (43.87) | 1.0 |
| 1 | 2171 (56.13) | 1.05 (0.88, 1.26) 0.609 |
| Ethnicity, n (%) |  |  |
| 0 | 2772 (71.66) | 1.0 |
| 1 | 399 (10.32) | 0.90 (0.66, 1.22) 0.495 |
| 2 | 697 (18.02) | 0.93 (0.73, 1.18) 0.544 |
| Vital signs |  |  |
| Heartrate, beats/min | 89.47 ± 16.81 | 1.01 (1.00, 1.01) 0.005 |
| SBP, mmHg | 115.27 ± 16.43 | 1.01 (1.01, 1.02) <0.001 |
| DBP, mmHg | 59.77 ± 10.66 | 1.02 (1.01, 1.03) <0.001 |
| MBP, mmHg | 76.49 ± 10.98 | 1.02 (1.01, 1.02) <0.001 |
| RR, times/min | 20.04 ± 4.47 | 1.03 (1.01, 1.05) 0.003 |
| Temperature, ℃ | 36.85 ± 0.72 | 1.18 (1.04, 1.34) 0.011 |
| SpO2, % | 97.15 ± 2.42 | 0.99 (0.96, 1.03) 0.636 |
| Comorbidities, n (%) |  |  |
| CS |  |  |
| No | 3673 (94.96) | 1.0 |
| Yes | 195 (5.04) | 0.70 (0.44, 1.11) 0.128 |
| CHF |  |  |
| No | 2580 (66.70) | 1.0 |
| Yes | 1288 (33.30) | 1.12 (0.93, 1.35) 0.251 |
| Cardiac arrhythmias |  |  |
| No | 2562 (66.24) | 1.0 |
| Yes | 1306 (33.76) | 1.11 (0.92, 1.34) 0.278 |
| PCD |  |  |
| No | 3503 (90.56) | 1.0 |
| Yes | 365 (9.44) | 1.07 (0.80, 1.45) 0.643 |
| Valvular disease |  |  |
| No | 3325 (85.96) | 1.0 |
| Yes | 543 (14.04) | 0.97 (0.75, 1.25) 0.803 |
| PVD |  |  |
| No | 3407 (88.08) | 1.0 |
| Yes | 461 (11.92) | 1.41 (1.10, 1.82) 0.008 |
| Hypertension |  |  |
| No | 1661 (42.94) | 1.0 |
| Yes | 2207 (57.06) | 1.13 (0.94, 1.36) 0.187 |
| Diabetes |  |  |
| No | 3016 (77.97) | 1.0 |
| Yes | 852 (22.03) | 0.86 (0.69, 1.08) 0.195 |
| Renal failure |  |  |
| No | 3055 (78.98) | 1.0 |
| Yes | 813 (21.02) | 1.20 (0.97, 1.48) 0.096 |
| Liver disease |  |  |
| No | 3385 (87.51) | 1.0 |
| Yes | 483 (12.49) | 0.71 (0.53, 0.96) 0.026 |
| Drug abuse |  |  |
| No | 3704 (95.76) | 1.0 |
| Yes | 164 (4.24) | 2.82 (2.00, 3.98) <0.001 |
| Alcohol abuse |  |  |
| No | 3525 (91.13) | 1.0 |
| Yes | 343 (8.87) | 2.18 (1.68, 2.84) <0.001 |
| Psychoses |  |  |
| No | 3679 (95.11) | 1.0 |
| Yes | 189 (4.89) | 2.43 (1.74, 3.38) <0.001 |
| Laboratory parameters |  |  |
| MLR | 0.61 ± 0.97 | 1.21 (1.02, 1.43) 0.026 |
| MLR tertiles, n (%) |  |  |
| <0.27 | 1288 (33.30) | 1.0 |
| ≥0.27, ≤0.53 | 1288 (33.30) | 1.44 (1.15, 1.81) 0.001 |
| >0.53 | 1292 (33.40) | 1.44 (1.15, 1.80) 0.002 |
| Neutrophils, % | 78.80 ± 14.62 | 1.00 (1.00, 1.01) 0.242 |
| Lymphocytes, % | 12.22 ± 10.31 | 0.98 (0.97, 0.99) 0.002 |
| WBC, 10^9^/L | 13.50 ± 10.71 | 0.99 (0.98, 1.00) 0.279 |
| Mean glucose, mg/dl | 143.44 ± 45.18 | 1.00 (1.00, 1.00) 0.577 |
| Monocyte, % | 4.29 ± 3.50 | 1.02 (0.99, 1.04) 0.128 |
| Anion gap, mmol/L | 16.95 ± 5.53 | 1.02 (1.00, 1.03) 0.029 |
| Albumin, mg/dl | 3.07 ± 0.70 | 1.04 (0.88, 1.23) 0.635 |
| Bilirubin, mg/dl | 2.39 ± 5.26 | 0.98 (0.96, 1.00) 0.118 |
| Creatinine, mg/dl | 1.99 ± 2.13 | 1.03 (0.99, 1.07) 0.101 |
| Chloride, mmol/L | 108.14 ± 7.11 | 1.00 (0.99, 1.01) 0.981 |
| Maximum glucose, mg/dl | 192.00 ± 103.55 | 1.00 (1.00, 1.00) 0.793 |
| Hematocrit, % | 35.53 ± 6.15 | 1.00 (0.99, 1.01) 0.958 |
| Hemoglobin, g/dl | 11.78 ± 2.12 | 1.00 (0.95, 1.04) 0.824 |
| Lactate, mmol/L | 3.35 ± 2.80 | 0.96 (0.93, 1.00) 0.044 |
| Potassium, mmol/L | 4.86 ± 0.99 | 0.95 (0.86, 1.04) 0.254 |
| APTT, second | 48.46 ± 32.86 | 1.00 (0.99, 1.00) 0.017 |
| INR | 1.83 ± 1.69 | 1.00 (0.95, 1.05) 0.948 |
| PT, second | 18.65 ± 11.88 | 1.00 (1.00, 1.01) 0.487 |
| Sodium, mmol/L | 140.53 ± 5.27 | 1.01 (0.99, 1.03) 0.208 |
| BUN, mg/dl | 35.46 ± 27.84 | 1.00 (1.00, 1.01) 0.085 |
| Bicarbonate, mmol/L | 24.61 ± 4.96 | 1.01 (0.99, 1.03) 0.234 |
| Band neutrophils, % | 11.03 ± 11.34 | 1.00 (0.99, 1.01) 0.969 |
| Scoring system |  |  |
| ECI | 19.07 ± 14.27 | 1.02 (1.01, 1.02) <0.001 |
| SOFA | 6.22 ± 3.75 | 1.03 (1.00, 1.05) 0.024 |
| SAPSII | 42.58 ± 14.78 | 1.01 (1.00, 1.01) 0.0267 |

Abbreviations: OR: odds ratio; CI: confidence interval; SBP: systolic blood pressure; DBP: diastolic blood pressure; MBP: mean blood pressure; RR: respiration rate; SpO2: pulse oximetry-derived oxygen saturation; CS: cardiac shock; CHF: congestive heart failure; PCD: pulmonary circulation disease; PVD: peripheral vascular disease; WBC: white blood cell; APTT: activated partial thromboplastin time; PT: prothrombin time; INR: international normalized ratio; BUN: blood urea nitrogen; ECI: Elixhauser comorbidity index; SOFA: sequential organ failure assessment; SAPSII: simplified acute physiology score II.
